# DNA-Encoded Chromatin Structural Intron Boundary Signals Identify Conserved Genes with Common Function

**DOI:** 10.1155/2015/167578

**Published:** 2015-03-11

**Authors:** Justin A. Fincher, Gary S. Tyson, Jonathan H. Dennis

**Affiliations:** ^1^Department of Computer Science, Florida State University, Tallahassee, FL 32306, USA; ^2^Department of Biological Science, Florida State University, Tallahassee, FL 32306, USA

## Abstract

The regulation of metazoan gene expression occurs in part by pre-mRNA splicing into mature RNAs. Signals affecting the efficiency and specificity with which introns are removed have not been completely elucidated. Splicing likely occurs cotranscriptionally, with chromatin structure playing a key regulatory role. We calculated DNA encoded nucleosome occupancy likelihood (NOL) scores at the boundaries between introns and exons across five metazoan species. We found that (i) NOL scores reveal a sequence-based feature at the introns on both sides of the intron-exon boundary; (ii) this feature is not part of any recognizable consensus sequence; (iii) this feature is conserved throughout metazoa; (iv) this feature is enriched in genes sharing similar functions: ATPase activity, ATP binding, helicase activity, and motor activity; (v) genes with these functions exhibit different genomic characteristics; (vi) *in vivo* nucleosome positioning data confirm ontological enrichment at this feature; and (vii) genes with this feature exhibit unique dinucleotide distributions at the intron-exon boundary. The NOL scores point toward a physical property of DNA that may play a role in the mechanism of pre-mRNA splicing. These results provide a foundation for identification of a new set of regulatory DNA elements involved in splicing regulation.

## 1. Background

### 1.1. Introduction

Eukaryotic gene expression is controlled at multiple levels, and splicing of mRNA is an important regulatory step in the production of functional proteins. During mRNA splicing, portions of the RNA, introns, are removed by the spliceosome complex, and the remaining protein-coding RNAs, exons, are joined together [1]. Alternative splicing, in which different combinations of exons are included in the final protein-coding mRNA, is responsible for the diversity of protein-coding mRNAs that can be derived from a single open reading frame [1–4]. The location of these splice junctions is generally defined by a G-T DNA sequence signal at the splice donor and an A-G DNA sequence signal at the splice acceptor [1, 5]. It is likely that more subtle sequence features in addition to the local sequence context of these splice sequences play a role in both constitutive splicing and alternative splicing. Transcription and splicing appear to be mechanistically coupled [6–14], and the precise rules governing recognition and regulation of constitutive and alternative splicing are poorly understood. Several recent papers have focused on the role that the packaging of the template DNA into chromatin plays in the splicing process [6, 15–23].

The packaging of eukaryotic DNA into chromatin is expected to affect all DNA templated processes. The fundamental subunit of chromatin is the nucleosome, 150 base pairs (bp) of DNA wrapped around a histone octamer. The position and density of nucleosomes play key regulatory roles and are controlled both by chromatin regulatory complexes and by features intrinsic to the DNA sequence [24–26]. There is limited information on how these two determinants of nucleosome occupancy coordinate to regulate responses, such as transcription in mammalian cells.

Nucleosome forming and nucleosome inhibitory properties were derived from first principles more than three decades ago [27]. More recently, maps of nucleosome distribution have enabled the development of computational models that use DNA sequence features to predict the nucleosome forming or inhibitory potential of DNA. Position specific scoring matrices [26] were developed by aligning strong nucleosome forming sequences and identifying the positions of enriched dinucleotide composition. Machine learning tools [24, 28], such as support vector machines (SVMs), have been used to predict the intrinsic nucleosome occupancy likelihood (NOL) for any DNA sequence. The support vector machine used in these studies is discriminative, rather than generative, and uses DNA sequences representing the strongest and the weakest affinity for forming nucleosomes. In these studies, DNA sequences protected from MNase digestion were identified with tiling microarrays [29, 30]. Genomic loci with the highest and the lowest nucleosome occupancy signals were used to train an SVM. The resulting algorithm can be used to classify the nucleosome forming potential of any genomic sequence [24]. A comparative assessment of available nucleosome occupancy prediction algorithms revealed that the SVM trained on human chromatin worked well on related species with relatively large, complex genomes [31]. These seminal discoveries affirmed an important role for intrinsic DNA sequence features influencing chromatin organization and revealed the utility of combined genomics and computational approaches for chromatin research.

Nucleosome occupancy has recently been shown to play a regulatory role at exon boundaries. These exonic nucleosomes have been proposed to act as “speed bumps” that allow for cotranscriptional splicing [32, 33]. Additionally, posttranslational modifications of the nucleosomal histones at these exonic boundaries have been shown to affect splice site usage [18, 19, 34]. This exonic nucleosomal occupancy is conserved throughout metazoan evolution [20], indicating a role for genomic sequence in this process.

### 1.2. Approach

We have previously described a computational model of nucleosomal occupancy trained on DNA sequence content [24]. A large fraction of nucleosome positions can be accurately predicted based on DNA sequence, indicating a significant DNA sequence component to nucleosome positioning [25, 26]. Nucleosome positioning signals at exon boundaries are conserved throughout evolution, and we reasoned that the analysis of predicted DNA-encoded nucleosomal occupancy would improve characterization of regulatory features associated with intron-exon boundaries. We used predictions of nucleosome occupancy to characterize intron/exon boundaries in five metazoan species and identified a cryptic sequence-based DNA property that is specific to a subset of fundamental metabolic genes. Our results suggest that, by defining regulated positions for nucleosomes, DNA features other than the consensus splice site sequence play a role in splicing.

## 2. Methods

### 2.1. DNA Sequences and Annotations

The DNA sequences for the current builds of all organisms (human, hg19; rat, rn4; zebrafish, danRer7; fly, dm3; worm, ce10; yeast, sacCer3) in this analysis were downloaded from the UCSC Genome Bioinformatics website (http://hgdownload.cse.ucsc.edu/downloads.html). Annotations for gene structure (transcription start site, strand, and boundaries between introns and exons) were acquired from the refGene tables of the UCSC Genome Browser public MySQL database with the exception of yeast for which the sgdGene table was used.

Nucleosome occupancy likelihood (NOL) scores were generated using the support vector machine (SVM) model derived from the Ozsolak A375 dataset (described in [24]). The SVM scores a 50 bp window of DNA sequence and uses a 1 bp step sliding window for sequences longer than 50 bp. The resulting score indicates the likelihood that the associated 50-mer is nucleosome forming (positive value) or nucleosome inhibitory (negative value).

### 2.2. Gene Ontology Analysis

Gene ontology analysis was completed with the GOrilla software [35] using genes of interest in the target set and the list of all genes in the associated genome as the background set.

### 2.3. Statistical Analysis

For calculations of significance across genomic statistics in enriched ontological categories, outliers were first excluded. Outliers were defined as those values less than the first quartile minus the interquartile range (IQR) or greater than the third quartile plus the IQR. *P* values were then calculated using a random sample of the same size from the whole genome and all values from the set of interest to perform a two sample *t*-test with a *P* value of <0.05 being considered significant.

## 3. Results and Discussion

### 3.1. NOL Plots Identify a Pattern of Nucleosomal Occupancy at Intron-Exon Boundaries

**Figure 1:**
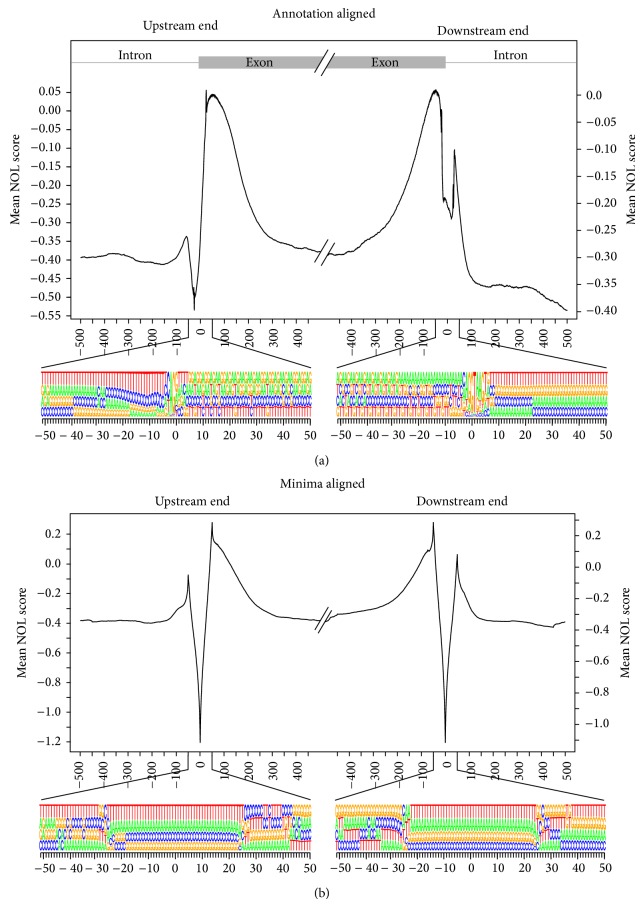
Mean nucleosome occupancy likelihood (NOL) scores for aligned sequences at the boundaries between introns and exons. Mean nucleosome occupancy likelihood (NOL) scores for aligned sequences at the boundaries between introns and exons. (a) Mean NOL scores for the regions +/−500 bp from the annotated upstream end and downstream end of the exons. For the central region +/−50 bp, nucleotide representation at each position is indicated by the size of the letters. (b) Mean NOL scores and associated nucleotide representations for the regions centered on the minimum value found within +/−50 bp of the annotated boundary between intron and exon.

We were first interested in discovering if there was any intrinsic nucleosome occupancy information in the regions flanking intron-exon boundaries. We reasoned that if chromatin structure plays a role in cotranscriptional splicing, then a robust location to store that chromatin structural information would be within the DNA sequence itself. To this end, we retrieved all intron-exon boundaries from the RefSeq annotation of the human genome [36]. We calculated nucleosome occupancy likelihood (NOL) scores for sequences spanning 1000 bp centered on intron-exon boundaries. NOL scores were calculated for each 50-mer and the resulting value was assigned to the center nucleotide position. NOL scores were plotted to reveal overarching trends of predicted nucleosome positions at intron-exon boundaries. Consistent with previous observations, we detected characteristic nucleosome positions at these boundaries (Figure 1(a)). Sequence analysis of the regions surrounding these boundaries showed clear consensus splice donor and splice acceptor sequences (Figure 1(a)). Closer inspection of the plots shown in Figure 1(a) revealed a sharp dip in average NOL scores for the introns both upstream and downstream of exons. This sharp dip, identified by the NOL, could only result from a set of sequence features that reduce the nucleosome forming potential at that location. We were next interested in understanding the characteristics of the sequences that contributed to this sharp dip.

### 3.2. Nucleosome Occupancy at Intron-Exon Boundaries Identifies a Characteristic Subpattern Feature

We hypothesized that the dip in the NOL scores may represent a functional DNA-encoded chromatin-regulatory structural element, as this is what the NOL plots measure. To investigate this possibility, we aligned the entire dataset to this putative regulatory feature by centering each region on the minimum found in the 100 base pairs centered on the boundary (Figure 1(b)). The percentage representation of each base at all positions was calculated and graphically represented to identify a previously undetected consensus sequence defining intron-exon boundaries. The sequence composition of these shifted subsets reflected equal representation of all four DNA bases and therefore did not reveal any clear consensus sequence feature (Figure 1(b), sequence diagram). This result suggests that a more cryptic combination of sequence features reflecting some physical property of DNA may be defining this low scoring element.

**Figure 2:**
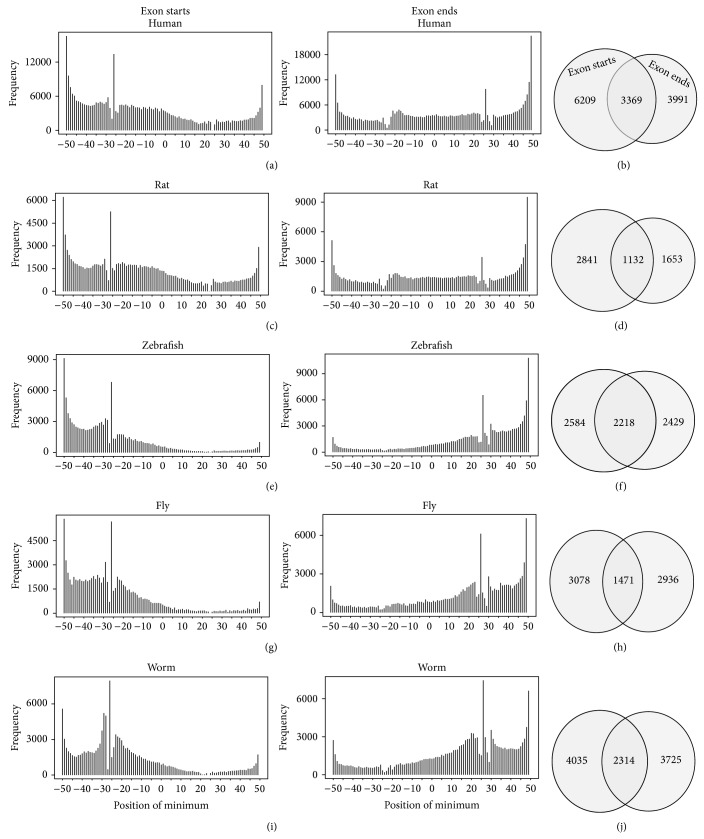
Counts of exons and genes grouped by location of minimum NOL score in the region +/−50 bp from the annotated boundary between intron and exon. Counts of exons and genes grouped by location of minimum NOL score in the region +/−50 bp from the annotated boundary between intron and exon. ((a), (c), (e), (g), and (i)) Histograms for human, rat, zebrafish, fly, and worm showing the counts of minimum values at each position in the region +/−50 bp from the annotated intron/exon (exon starts) and exon/intron (exon ends) boundaries. ((b), (d), (f), (h), and (j)) Venn diagrams indicating the numbers of genes represented in the −26 peak for exon starts and +26 peak for exon ends and the overlap between the two sets.

Different biophysical properties emerge with different DNA sequence combinations (e.g., DNA wedge angles [37]). We were, therefore, interested in determining if the dips upstream and downstream conferred by the DNA sequence of the exon might identify a cryptic characteristic of intron boundary architecture. We calculated the location of the minimum NOL score for each boundary window and then represented these data as a histogram of distance of the minima from the intron-exon boundary (Figure 2(a)). We observed a striking overrepresentation −26 nucleotides (nt) from the annotated intron-exon boundary, upstream of the exon (U − 26). We also found a similar feature +26 nt from the exon-intron boundary, downstream of the exon (D + 26). The discovery of the U − 26 and D + 26 features prompted us to investigate how many and what types of genes include these features.

In order to understand the numbers and types of genes associated with the U − 26 and D + 26 feature, we selected the exons represented in each of these groups, U − 26 and D + 26, for further analysis. There are 9578 genes represented in the U − 26 group and 7360 genes represented in the D + 26 group, representing 24.1% and 18.5% of all open reading frames tested, respectively. We next wanted to know if the U − 26 and D + 26 features were found in the same sets or different sets of genes. 3369 genes, or 19%, overlap between the U − 26 and D + 26 groups (Figure 2(b)).

### 3.3. The Intron-Exon Boundary Feature Is Conserved across Metazoa

As positioned nucleosomes flanking exons are phylogenetically conserved, we were next interested in determining whether the prominent U − 26 and the D + 26 signature are conserved in other metazoan species. Conservation of these features would suggest an important role for the U − 26 and the D + 26 causing it to be maintained by evolution. We compared wide ranging species including rat, zebrafish, fly, and worm. We identified the nucleotide position of the minimum at the boundary between intron and exon for rat, zebrafish, fly, and worm. A conspicuous U − 26 and D + 26 signature exists for all of these species (Figures 2(c), 2(e), 2(g), and 2(i)). As with the human example above, we identified substantial overlap for the genes containing this signature. This result led us to hypothesize that the signature was associated with a particular gene set that is conserved across each of the species tested.

### 3.4. The U − 26 and the D + 26 Signatures Identify Specific Groups of Ontologic Function

The conservation of these features suggested a role in genomic regulation. We next wanted to identify the feature that is present in groups of genes with related function. In order to test whether the U − 26 or the D + 26 signatures identified groups of genes that share a common function, we searched for ontological enrichment [35]. The U − 26 and the D + 26 signatures both showed enrichment for overlapping groups of gene function (Table 1). These groups include ATPase activity, ATP binding, helicase activity, and motor activity. With little exception, these enrichments persist throughout all metazoan species tested (Table 1). These results led us to hypothesize that these groups of genes contain genomic characteristics that differ from the set of all genes.

**Figure 3:**
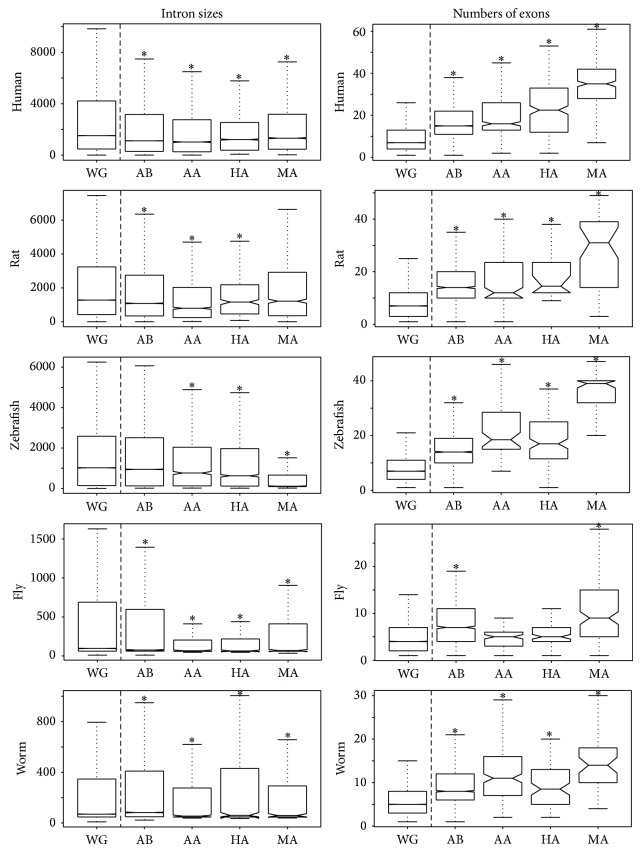
Boxplots of genomic characteristics for several ontological categories in comparison to the entire genome. Boxplots of intron sizes and numbers of exons are shown for the whole genome (WG), ATP binding (AB), ATPase activity (AA), helicase activity (HA), and motor activity (MA) across the 5 species of interest. Values that differ significantly from the whole genome are indicated with an asterisk.

In order to test whether genes found in the enriched functional categories containing the U − 26 and the D + 26 signature had genomic characteristics that varied significantly from the rest of the genome, we compared exon size, intron size, and number of exons for each function category to the same values calculated for the genome as a whole (Figure 3). We were able to identify trends for each of these functional categories. The exon sizes of genes included in each of the categories did not differ substantially from the exon sizes encoded by each genome (Supplementary Figure  1, available online at http://dx.doi.org/10.1155/2015/167578). Intron sizes of the genes in the ontological function categories were significantly shorter when compared to the respective whole genomes with few exceptions (rat: motor activity, zebrafish: ATP binding, and worm: ATP binding). Across all organisms, with the exception of ATPase activity and helicase activity in rat, all ontological categories across all organisms exhibited significantly higher numbers of exons per gene. For example, the human genome has a median of seven exons per gene while the subset of human motor activity genes has approximately five times the number of exons with a median of 35 exons per gene.

### 3.5. *In Vivo* Nucleosome Maps Show That Loci Containing U − 26 and the D + 26 Signatures Are Enriched for Specific Ontologic Function

As our experiments, to this point, had been purely based on in silico experiments and genome sequence data, we wanted to see how our results compared to published* in vivo* data. We next were most interested in testing if the U − 26 or the D + 26 signatures are observed* in vivo*. We used the nucleosome maps generated by the ENCODE consortium for the two cell lines, GM12878 and K562. We retrieved all nucleosome measurements for intron-exon boundaries for each of the two cell lines from http://genome.ucsc.edu/ [36] and identified the location of the minimum within the central 100 base pairs surrounding intron-exon boundaries as described previously using the NOL scores. Approximately 2.5% of genes contained intron-exon boundaries exhibiting the D − 26 and U + 26 characteristic (Table 2). We wanted to know whether this* in vivo* signature was associated with the shared common function consistent with those identified from the NOL predictions. Three of the four categories identified by the NOL score signal, ATP binding, ATPase activity, and motor activity, were also significantly enriched in the* in vivo* data. Random samples of similar numbers of genes showed no ontological enrichment. These findings indicate that sequence-based nucleosome forming signals at the boundaries between introns and exons may play a role in the regulation of these genes.

### 3.6. Loci Containing U − 26 Feature Have Distinctive Differential Intronic and Exonic Dinucleotide Content

**Figure 4:**
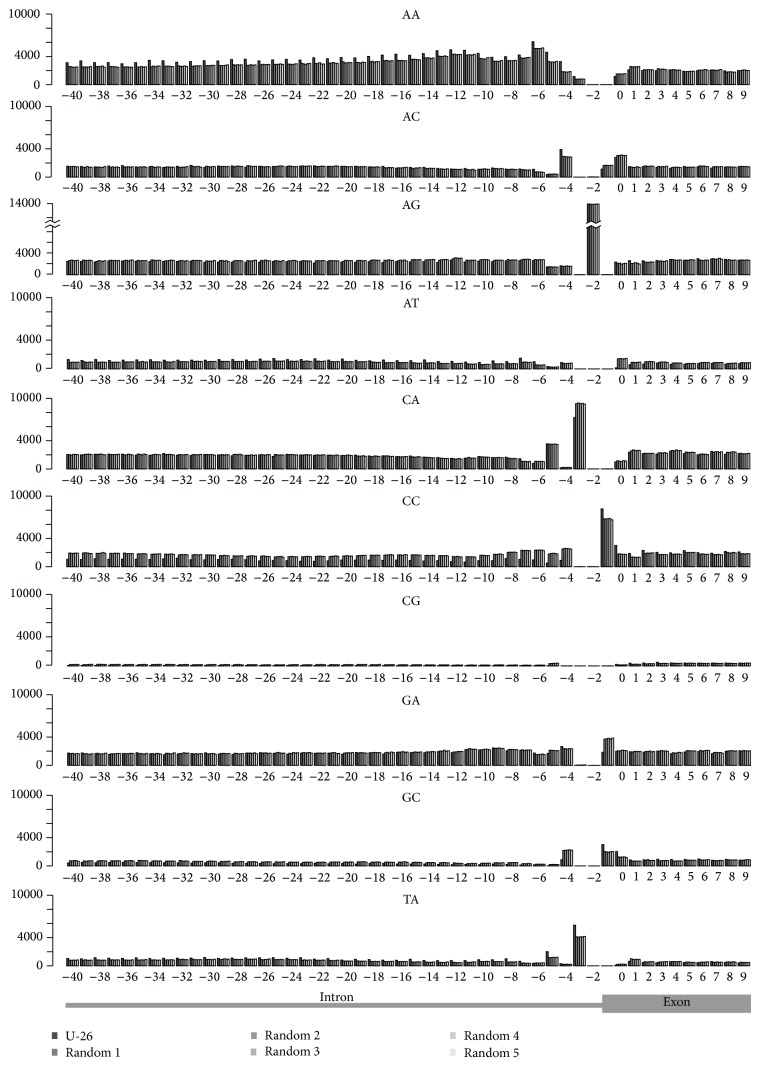
Dinucleotide counts at intron-exon boundary. The dinucleotide counts for the region −40 to +10 nucleotides from the upstream boundary between intron and exon as indicated at the bottom of the figure. Solid black bar indicates the counts for exons containing the U − 26 signal. Grey bars represent 5 random samples of similar size for comparison.

The upstream intron contains a region between the branchpoint sequence and the 3′ splice site that is generally depleted of AG dinucleotides. This region is generally within 40 nucleotides of the AG splice site and encompasses the U − 26 feature. We were interested in determining if the loci containing the U − 26 feature were enriched or depleted for any dinucleotide occurrences relative to the rest of the genome. We calculated dinucleotide frequency for the ten dinucleotides for the loci containing the U − 26 feature and compared that to the dinucleotide frequency for an equal number of other intron-exon boundaries in the genome (Figure 4). We found that the intronic region off these genes is actually slightly more depleted of AG dinucleotides than other intron-exon boundaries. We were next interested in determining if the remaining dinucleotides showed differential intron and exon content.

Recent work has shown that differential G/C content plays a role in the intron exon definition and splice site selection [14, 38]. CC, CG, and GC dinucleotides were depleted in introns of the loci containing the U − 26 feature. Likewise, AA, AT, and TA dinucleotides were enriched in the introns of the loci containing the U − 26 feature compared to equivalent regions in the rest of the genome (Figure 4). Loci containing the U − 26 feature have a lower overall intronic G/C content. The exonic region, however, shows the opposite trend with decreased A/T and increased G/C content. The CC dinucleotide feature most strongly defines this set of loci and is depleted from introns and enriched in exons. This would suggest that dinucleotide content plays a larger role in the definition of intron-exon boundary than was previously anticipated.

## 4. Conclusion

We have identified a set of conserved genes sharing a common function using a nucleosome positioning signature. We have further characterized the set of genes as having increased numbers of exons while having average number and length of introns. This feature has been validated using* in vivo* mapped nucleosome positions. Finally, we have shown that unique dinucleotide content distinguishes this set of loci. Thus, we have identified a set of conserved genes with common function and distinguishing features that suggest shared regulation.

Our results indicate that cryptic sequence features may drive DNA-templated regulatory events. Our observations and the classification of a particular subset of genes could not have been accomplished through alignment of nucleotide content. The NOL scores point toward a physical property of DNA related to the ability of a particular DNA sequence to form a nucleosome. The organization and architecture of DNA around the nucleosome may likely play a role in the mechanism of pre-mRNA splicing. We anticipate that many more functional DNA elements may be discovered using similar methodologies.

## Supplementary Material

Boxplots of exon sizes for several ontological categories in comparison to the entire genome. Boxplots are shown for the whole genome (WG), ATP Binding (AB), ATPase Activity (AA), Helicase Activity (HA), and Motor Activity (MA) across the 5 species of interest. Values that differ significantly from the whole genome are indicated with an asterisk.

## Figures and Tables

**Table 1:** *P* values indicating significance of ontological enrichment across species.

Organism-region	ATPase activity	ATP binding	Helicase activity	Motor activity
Human U − 26	3.84*e* ^−17^	<1*e* ^−34^	2.98*e* ^−11^	1.38*e* ^−16^
Human D + 26	2.64*e* ^−16^	2.32*e* ^−29^	3.0*e* ^−9^	1.53*e* ^−9^

Rat U − 26	4.67*e* ^−11^	7.92*e* ^−34^	4.43*e* ^−7^	1.22*e* ^−15^
Rat D + 26	7.84*e* ^−11^	1.2*e* ^−20^	2.8*e* ^−7^	1.17*e* ^−5^

Zebrafish U − 26	8.83*e* ^−10^	2.85*e* ^−33^	9.61*e* ^−9^	2.82*e* ^−10^
Zebrafish D + 26	4.77*e* ^−16^	2.79*e* ^−28^	1.14*e* ^−7^	1.04*e* ^−7^

Fly U − 26	3.72*e* ^−5^	8.3*e* ^−11^	N/A	3.99*e* ^−4^
Fly D + 26	N/A	3.83*e* ^−7^	N/A	4.27*e* ^−6^

Worm U − 26	2.07*e* ^−4^	4.83*e* ^−20^	3.7*e* ^−4^	1.89*e* ^−6^
Worm D + 26	7.81*e* ^−10^	9.28*e* ^−21^	3.36*e* ^−5^	3.25*e* ^−5^

Measures of significance of enrichment as indicated by GOrilla [35]. Values are shown for intron/exon (U − 26) and exon/intron (D + 26) across 5 species.

**Table 2:** *P* values indicating significance of ontological enrichment for genes exhibiting the U − 26 or D + 26 feature *in vivo*.

GO category	K U − 26	G U − 26	K D + 26	G D + 26
ATP binding	8.86*e* ^−07^	N/A	4.04*e* ^−05^	8.55*e* ^−05^
ATPase activity	9.32*e* ^−04^	N/A	N/A	N/A
Helicase activity	N/A	N/A	N/A	N/A
Motor activity	N/A	6.76*e* ^−04^	N/A	2.47*e* ^−05^
Number of genes	587	655	647	636

*P* values indicating significance of ontological enrichment for genes exhibiting the U − 26 or D + 26 feature *in vivo* in two cell lines, K562 (K) and Gm12878 (G). Measures of significance of enrichment as indicated by GOrilla [35].
