# Supplementary figures and images for: DNA-Encoded Chromatin Structural Intron Boundary Signals Identify Conserved Genes with Common Function
Source: Int J Genomics. 2015 Mar 11;2015:167578. doi: 10.1155/2015/167578 (PMC4377520; doi:10.1155/2015/167578)

## Exon Sizes

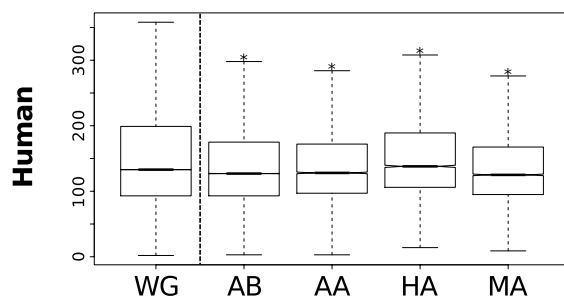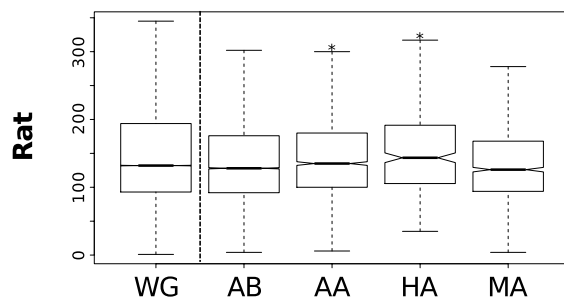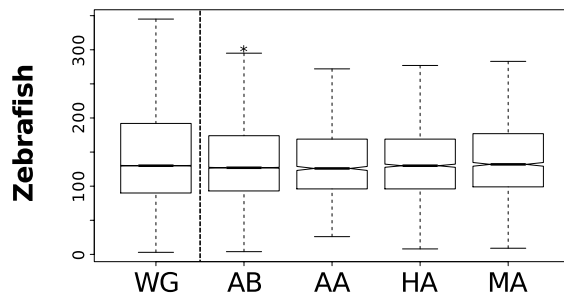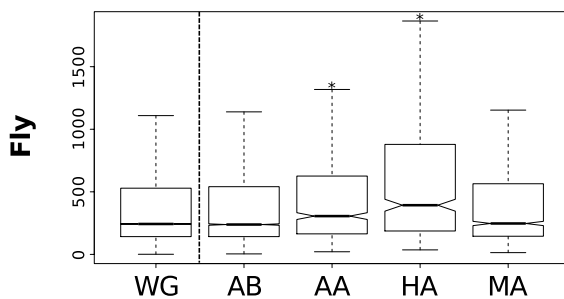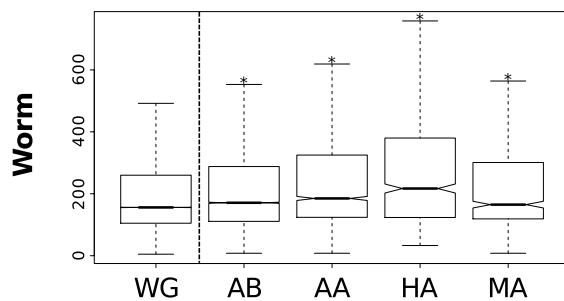

Supplement: Supplementary file 1 — Boxplots of exon sizes for several ontological categories in comparison to the entire genome. Boxplots are shown for the whole genome (WG), ATP Binding (AB), ATPase Activity (AA), Helicase Activity (HA), and Motor Activity (MA) across the 5 species of interest. Values that differ significantly from the whole genome are indicated with an asterisk. [file 167578.f1.pdf]
